# Anatomic Region of Cutaneous Melanoma Impacts Survival and Clinical Outcomes: A Population-Based Analysis

**DOI:** 10.3390/cancers15041229

**Published:** 2023-02-15

**Authors:** Christian M. Shannon, Neil K. Mehta, Hong Li, Shaun A. Nguyen, Sina Koochakzadeh, Dirk M. Elston, John M. Kaczmar, Terry A. Day

**Affiliations:** 1 Department of Otolaryngology, Medical University of South Carolina, Charleston, SC 29425, USA; 2 Department of Otolaryngology, The Ohio State University College of Medicine, Columbus, OH 43210, USA; 3 Division of Biostatistics, Davis School of Medicine, University of California, Davis, CA 95616, USA; 4 Department of Otolaryngology, University of Florida College of Medicine, Gainesville, FL 32610, USA; 5 Department of Dermatology, Medical University of South Carolina, Charleston, SC 29425, USA; 6 Sarah Cannon Head & Neck Oncologic and Reconstructive Surgery, Charleston, SC 29406, USA

**Keywords:** cutaneous melanoma, epidemiology, survival, anatomical site, cancer

## Abstract

**Simple Summary:**

Despite the increasing incidence of melanoma in the United States, few studies have compared tumor and clinical characteristics of cutaneous melanoma by anatomic region with an analysis of survival outcomes. The goal of the current study was to determine how the anatomic region of a cutaneous melanoma affects an individual’s overall survival rate. In this cross-sectional study that included 178,892 cases, cutaneous melanoma of the head and neck region was associated with the greatest risk of death (HR 1.90 [95% CI, 1.85–1.96]) compared to other sites, a finding that suggests that anatomic site should be considered for inclusion in future editions of staging criteria to improve the overall management of patients diagnosed with cutaneous melanoma.

**Abstract:**

Purpose: The objective was to determine the effects of the anatomic site of a cutaneous melanoma on the survival outcomes of diagnosed individuals. Methods: We conducted a cross-sectional study using data from the Surveillance, Epidemiology, and End Results Program (SEER) Database from 2004–2014 and included 178,892 cases of individuals diagnosed with cutaneous melanoma. Overall survival (OS) for each anatomic site as well as associated demographics, primary site, stage, and pathologic prognostic factors (Breslow’s depth of invasion (DOI), level of mitoses, and ulceration), were analyzed. Results: Lower extremity melanoma (LEM) was the most likely to have locoregional nodal spread, yet head and neck melanoma (HNM) was the most likely to present at the most advanced stage of disease (IV). Independent of other factors, HNM was associated with the greatest risk of death (HR 1.90 [95% CI, 1.85–1.96]) compared to other sites, and males experienced worse overall survival (OS) (HR 1.74 [95% CI, 1.70–1.78]) compared to females. The last and greatest risk of death is associated with LEM and HNM, respectively. Conclusion: Given these survival differences, consideration should be given to incorporating the primary site of melanoma into staging to ensure treatment is efficacious as possible.

## 1. Introduction

Melanoma represents nearly 1% of skin cancer diagnoses and is responsible for the majority of deaths related to cutaneous malignancies [1]. In the United States, the incidence of melanoma has increased over the previous two decades, with a projected 99,780 new cases to be diagnosed in 2022 [1]. This has occurred despite the increasing utilization of sun-blocking agents, as demonstrated by the CDC report that 70.8% of US adults in 2020 used sun-protection [2,3]. However, mortality from melanoma has also declined over the last decade to a rate of 3.2% per year, which may be due to earlier diagnosis and continuously advancing treatment modalities [4]. 

In addition, an advanced understanding of prognostic factors is likely responsible for declining mortality. The work of pathologists, in particular, has led to the development of diagnostic criteria critical for the histological characterization of cutaneous melanoma [5]. Many well-established factors, including Breslow’s depth of invasion (DOI) as well as the degree of mitoses and ulceration, are associated with worse prognoses [6,7]. Despite this, the impact of the anatomic region on prognosis has yet to be established despite evolving guidelines and standards of care for melanoma. Cancer centers are increasingly organizing melanoma-based teams and multidisciplinary care from a variety of specialists, such as dermatologists, oncologists, plastic surgeons, pathologists, and otolaryngologists, to address the complexity of the disease. 

The primary site of cutaneous melanoma is often classified into one of four regions: head and neck melanoma (HNM), trunk melanoma (TM), upper extremities including shoulder melanoma (UEM), and lower extremities including hip melanoma (LEM). The trunk is most commonly affected (35–40%), with variable rates for the head and neck (15–30%) and extremities (15–40%) [8,9,10]. Our main objective was to assess survival trends in cutaneous melanoma related to the anatomic region of the primary lesion through a population-based database. Demographics, prognostic factors, and management were also evaluated.

## 2. Materials and Methods

Data for this study were obtained from the Surveillance, Epidemiology, and End Results Program (SEER) provided by the National Cancer Institute. This population-based database captures nearly 35% of new cancer diagnoses in the US. These registries contain pertinent information, including site, stage at diagnosis, initial treatment, and survival outcomes [11]. The SEER 18 Registry Research Data (cases from 2004 to 2014, released April 2017) was utilized with SEER*Stat software Version 8.3.4 [12]. 

### 2.1. Study Cohort

Patients were included based on a diagnosis of melanoma between 2004–2014, using the ICD-O-3 histology codes for cutaneous melanoma. This time period was specifically chosen to allow for an analysis of 5-year survival after the time of diagnosis. Patients were sorted based on the following primary location sites of melanoma: Head and Neck (External Upper Lip, External Lower Lip, External Lip Not Otherwise Specified [NOS], Lip NOS, Skin of Lip NOS, Eyelid, External Ear, Skin Unspecified Part of Face, and Skin Scalp/Neck), Trunk (chest, back, and abdomen), Upper Extremities/Shoulder, or Lower Extremities/Hip. Patients were excluded if there were duplicate melanoma cases with the same identifier or if the melanoma did not arise from the skin. Patients with multiple primary melanomas, as determined by their unique identifier, were excluded (*n* = 20,113). For Staging, the 6th and 7th editions of the American Joint Committee on Cancer (AJCC) staging system were utilized for patients diagnosed between 2004–2009 and 2010–2014, respectively [13]. 

### 2.2. Outcome Measures

The primary outcome assessed was overall survival (OS) for each anatomic subsite. Univariate and multivariate regressions were used to assess the relationship between melanoma location and survival outcomes. Additional data points assessed included demographics, primary site, stage, and pathologic prognostic factors (Breslow’s depth of invasion (DOI), level of mitoses, and ulceration). 

### 2.3. Statistics

Data analyses were performed with SAS 9.4 (SAS Institute INC., Cary, NC, USA) and SPSS 24.0 (SPSS Inc., IMB Corporation, Armonk, NY, USA). Categorical variables were summarized by frequency and percentage. Continuous variables were summarized by mean ± standard deviation (SD) or median and interquartile range (IQR) when appropriate. All continuous variables were assessed for normality using the Kolmogorov-Smirnov test. Comparisons of baseline characteristics and outcomes (categorical values) were performed using a Chi-Square test. For continuous variables, comparisons between three or more groups were made with a One-Way ANOVA/Kruskal Wallis test. To assess the effect of covariates on OS or death, a univariate cox regression analysis was performed first in which we fit cox regression models using each of the above-mentioned study variables at one time. The *p*-value less than or equal to 0.001 was used as the initial variable selection criteria. This *p*-value was selected given our patient population was ≥100,000 to ensure an appropriate sensitivity for this study. Variables with *p* < 0.001 in univariate Cox proportional hazards analysis were entered into a multivariate Cox proportional hazards model. The associations between variables and OS were evaluated by multivariable analysis utilizing the Cox proportional hazards model with the stepwise forward method with a significance level (PR) at 0.15 and with a significance level for addition to the model (PE) at 0.1 > 0.1 being criteria for exclusion for the final multivariable model. A final multivariate cox regression model with forward stepwise variable selection based on clinical and statistical importance was confirmed and was used to identify the final covariates which would have a significant impact on OS. A *p*-value of <0.05 was considered statistically significant for all statistical tests. In addition, Cohen’s d effect sizes were calculated with d = 0.2 is considered a ‘small’ effect size, 0.5 representing a ‘medium’ effect size, and 0.8 a ‘large’ effect size.

## 3. Results

### 3.1. Demographics

A total of 178,892 cases of cutaneous melanoma were identified between 2004–2014. Primary sites were distributed as such: HNM 21.4%, TM 33.7%, UEM 26.4%, and LEM 18.4%. The mean age was 60.6 years (SD 16.7), and the majority of patients were male (57%). HNM was most commonly found in older patients (mean age 66.6, SD 16.6) who were male (72%), whereas LEM was more commonly seen in younger patients (mean age 56.5, SD 16.8) who were female (70%). Nearly all patients (94.0%) identified their race as White. Among Black and Asian patients diagnosed with melanoma, the lower extremities were the most likely to be affected. Melanoma of any site was most commonly diagnosed in the Western region of the US (55% of total cases), and a majority of the cases from this geographical area affected the head and neck region (57%). Additional demographic data is outlined in Table 1.

### 3.2. Tumor Characteristics 

#### 3.2.1. Staging

The AJCC stage was available for 162,180 patients, with Stage I being the most common (71%). HNM was more frequently presented as Stage II (15.6%) or Stage IV (1.9%) compared to the other sites. LEM was the most likely to present with N+ disease, with 10.0% of cases Stage III or IV. The overall stage stratified by anatomic region is described in Table 2.

#### 3.2.2. Histology

The most common histology found was malignant melanoma NOS (50.2%), representing a significant confounder to cases collected. The second most common histology was superficial spreading melanoma (30.7%). Additional histological data can also be found in Table 2.

### 3.3. Management & Survival

#### 3.3.1. Management 

Patients that underwent surgical resection of the local tumor or gross excision had enhanced 5-year OS for all primary sites (Table 3). Surgery was most commonly performed for LEM. However, surgical management conferred the greatest increase in 5-year survival for HNM (mean difference +15% [95% CI, 10.0–20.2]), while all other anatomic regions only benefited 8.0% in 5-year survival rates. Sentinel lymph node biopsies (SLNB) were performed in 25% of total cases. This treatment was least commonly used for HNM (20% of cases) and most commonly used for LEM (30%). HNM managed with SLNB was associated with improved 5-year OS (mean difference +2.0% [95% CI, 0.1–3.9])), but SLNB was not associated with improved 5-year OS for the other sites (Table 3). Furthermore, multivariate regression demonstrated improved hazard ratios with SLNB (HR 0.55 [95% CI, 0.52 to 0.58]), indicating a 45% reduced risk of death (Table 4). 

For patients that underwent surgery, tumors were removed via local excision (13%), gross excision with <1 cm margins (48%), or gross excision with >1 mm margins (34%) (Table 5). Similar rates of local tumor excision were observed among all anatomic sites. HNM was most likely to undergo resection with <1.0 cm margins (51.3%). Gross excision with >1.0 cm margins was most frequently associated with TM (Table 5). 

#### 3.3.2. Pathologic Factors

The majority of cases were ≤1.00 mm in DOI (≥59%). HNM had the most advanced DOI compared to other sites, with 8.59% of cases > 4.00 mm (mean difference 2.74% [95%CI, 2.42–3.06]; *p* < 0.001). TM demonstrated the least advanced DOI, with 69.3% of cases between 0.0–1.00 mm. HNM also had the largest proportion of cases with mitoses present (18.9%). In contrast, the presence of melanoma with ulceration was most commonly found in LEM (14%) (Table 2). 

#### 3.3.3. Survival Analysis

Our patient cohort experienced an 80% 5-year survival for melanoma of any site. HNM had the worst outcomes (5-year OS 71%), and LEM had the best outcomes (5-year OS 85%). 2-year and 5-year OS outcomes by the primary site are outlined in Table 5. The qualitative presence of ulceration and mitoses were associated with reductions in 5-year OS rates by 30–38% and 13–27%, respectively. Mitoses had the greatest impact on HNM 5-year OS, while ulceration had the greatest impact on survival rates for LEM. Hazard ratios for tumor characteristics are described in Table 4. 

Upon controlling for covariates, we found the greatest risk of death for HNM (HR 1.90 [95% CI, 1.85–1.96]) and the least for LEM (HR 0.86 [95% CI, 0.82–0.89]). These survival outcomes were compared in reference to TM, which had similar survival outcomes as UEM (HR 1.06 [95% CI, 1.03–1.10]) (Table 4). In addition, age greater than 60 years had a considerable impact on the risk of death (HR 4.78 [95% CI, 4.64–4.92]), and male patients were more at risk compared to female patients (HR 1.74 [95% CI, 1.70–1.78]). 

## 4. Discussion

### 4.1. Survival 

Differences in survival between cutaneous melanoma sites have been previously studied. However, associated conclusions remain conflicting to date. For patients with early-stage disease (Stage I/II), the primary site was not associated with a difference in prognosis [14]. However, patient cohorts with the more advanced disease found HNM had a worse prognosis compared to other anatomic sites [8,15]. Our analysis indicates that the primary site is an important factor in prognosis: HNM was associated with the greatest hazard ratios compared to other sites, even after adjusting for other prognostic factors such as stage, DOI, and presence of ulceration or mitoses. This association was also supported in a similar analysis performed by Ding et al., which specifically looked at HNM vs. TM and found HNM cases had significantly lower rates of OS and cancer-specific survival (CSS) [16]. Therefore, discussions over survival and treatment should consider the primary site as a key prognostic factor. The lower rate of survival for this region could be attributed to conservative surgical margins due to aesthetic concerns, as well as less predictable lymphatic drainage patterns of the head and neck [17,18,19]. A population-based study focusing on the scalp region found this site to be associated with poorer 5-year and 10-year disease-specific survival, even after adjusting for other factors [20]. 

Differences in histopathological prognostic factors likely also played a role in survival differences. The presence of ulceration or mitoses was associated with survival differences in all anatomic regions, though the magnitude of 5-year survival differences was greater for ulceration. Therefore, refocusing the 8th AJCC criteria toward ulceration appears congruent with our analysis [21]. Ulceration had the most negative impact on survival for LEM, with a 38% reduction in 5-year OS, indicating evaluation for ulceration is most critical for this region. In the analysis performed by Ding et al., ulceration was also significantly associated with worse prognoses for both anatomic regions they included in their analysis, though they found a greater effect from ulceration on CSS for TM compared to HNM [16]. Meanwhile, mitoses had the greatest impact on HNM, associated with a 27% reduction in 5-year OS. While the mitotic rate was recently removed from the AJCC 8th staging criteria [21], our study suggests the presence of mitoses confers worse survival rates for all anatomic regions (HR 3.09 [95% CI, 2.91–3.27]). 

### 4.2. Demographics

HNM were more likely to present at an older age (mean 66.6) as well as in males, which are trends supported by the previous literature [16], while lesions of the lower extremities more frequently presented younger (mean 56.5) and in female patients. This finding confirms the results of previous studies associating HNM with males and older age at diagnosis [14,21,22]. This trend might be related to a chronic pattern of sun exposure to the head and neck over a lifetime [23,24]. High cumulative sun exposure places the head and neck at increased risk, per unit area, compared to other regions of the body, which receive comparatively intermittent sun exposure. This might also explain sex-related differences in survival observed in this study and others, as cumulative sun exposure could place males at greater risk of developing melanoma of the ears and scalp [25,26,27]. 

Furthermore, the Western region of the United States experienced the majority of total melanoma cases (55%). The warmer climate in this region, and therefore greater sun exposure with more time spent outside, might help explain this geographic distribution. Interestingly, only 8% of total cases were found in the Southern US despite a warmer climate there as well. The difference between the two regions is likely multifactorial and may include aspects such as comparatively limited access to healthcare, infrequent cancer screenings, or lower population density in the South. The discrepancy in the number of cases between the Western and Southern US regions also suggests that reporting practices to the SEER database may play a large role in melanoma geographic distributions. Because cases are typically reported from tertiary academic centers or national cancer centers located in urban regions, geographic distributions may skew towards certain areas, such as the east and west coasts, which have more cities [28]. 

### 4.3. Management

The therapeutic benefits of SLNB remain limited for many cases of cutaneous melanoma. Results from the Multicenter Selective Lymphadenectomy Trial (MSLT-I) demonstrated SLNB was not associated with improved disease-specific survival compared to observation alone [29]. Therefore, although SLNB has proven to be valuable for purposes of staging, the lack of established survival benefit has led to controversy regarding whether or not SLNB should be routinely performed. In our analysis, SLNB was most commonly performed for LEM (30% of cases) and least commonly for HNM (20% of cases). Interestingly, this site-specific data also correlated with both the best survival outcomes (LEM 5-year OS 85%) and worst survival outcomes (HNM 5-year OS 71%), indicating a possible contribution from SLNB to overall survival. 

Rates of SLNB likely correlate with rates of complete lymph node dissections (CLND). Therefore, it is possible that there were cases in which a CLND was not executed despite being indicated because an initial SLNB was never performed. The MSLT-II and DeCOG-SLT trials evaluated the efficacy of immediate lymphadenectomy compared to nodal observation for patients with positive SLNB and found no disease-specific survival benefit for CLND [30,31]. In our cohort, multivariate analysis on the performance of SLNB was associated with a decreased hazard ratio (HR 0.55 [95% CI, 0.52 to 0.58]), indicating improved survival. Therefore, although many cases of melanoma may not benefit from SLNB, our data demonstrate that if it is indicated and performed, it has the potential to significantly improve survival. Nonetheless, conclusions should be reached as these patients were treated at tertiary centers with access to multidisciplinary care and more novel treatments, such as immunotherapy which may not be ubiquitously available. 

In regards to surgical intervention, gross excision with wide margins was performed the least for HNM. Smaller margins and lower rates of excision for Stages I and II melanomas in the HNM location compared to other anatomic sites have been previously reported [14]. This may be related to additional anatomic considerations for the head and neck, with melanomas potentially close to critical areas such as the eyes, nose, or mouth, which are comparatively difficult to navigate. For patients with melanoma of HNM, Mohs micrographic surgery is often the best option, which, when performed, is associated with reduced hazard ratios compared to wide local excision [32]. Among all anatomic regions studied, HNM treated with either local tumor or gross excision had the greatest increase in 5-year OS (+15%) (Table 3). This may provide additional evidence to pursue surgery in cases of HNM when surgery is a consideration but not unequivocally indicated. Worse OS for patients who do not undergo surgery is likely related to significant comorbidities in this population. 

### 4.4. Staging

The AJCC revised its staging system in 2010, switching from the 6th to the 7th edition [13]. This transition occurred within the time frame of our data and included using mitoses instead of the level of invasion to define T1b melanomas [33]. Of note, the 8th edition of the AJCC staging system removed mitotic rate as a T-category criterion [34]. Instead, T1b melanomas were redefined as ulcerated lesions <1.0 mm thick or nonulcerated lesions 0.8–1.0 mm thick [21]. Future population-based analyses will need to assess the impact of these changes found in the 8th AJCC staging system. 

### 4.5. Limitations 

Patients with multiple melanomas (20,113) were excluded as their clinical data would be counted multiple times. Therefore, our conclusions cannot be applied to this subset of patients. In addition, several clinical factors identified in the previous literature as important for prognostication were unavailable in the SEER database, such as lymphovascular invasion, neurotropism, tumor-infiltrating lymphocytes, satellite lesions, and positive or negative SLNB [34]. Patient comorbidities played a key role in survival outcomes and were also not available through the SEER database. Lastly, data regarding the completion of lymphadenectomy or neck dissection were not available and should be assessed in future studies. 

We were also unable to account for the accurate number of patients that underwent chemotherapy and radiotherapy, as many were listed as “none/unknown”. Therefore, data for these two treatments were excluded. Immunotherapy was also not listed, so we were unable to evaluate the impact of this treatment modality. Given the significant impact immunotherapies have had on recurrence and survival outcomes for melanoma, this is an important limitation in our data set that should be addressed in the future. PD-1inhibitors have demonstrated improved recurrence-free survival as adjuvant treatment for Stage III melanoma and have been approved as adjuvant treatment for Stage IIB or IIC based on the results of Keynote-716 [35,36]. Finally, the data analyzed was derived from a retrospective national database with information that is subject to accurate coding and registration. 

## 5. Conclusions

Clinical and survival characteristics for cutaneous melanoma vary between anatomic sites. Melanoma most frequently presents on the trunk and upper extremities. Younger age (<60) was associated with tumors found on the lower extremity and trunk, while older age (≥60) was associated with tumors of the upper extremity and head and neck. LEM was the only primary site more frequently observed in females. Most patients with melanoma were White, though melanoma diagnosed in Black and Asian patients most frequently affected the lower extremities. HNM is associated with the worst 5-year survival outcomes, independent of prognostic factors such as DOI or degrees of ulceration or mitoses. Surgery provides survival benefits for all anatomic regions; however, this study suggests it may confer the greatest 5-year survival increase for HNM. Staging systems for melanoma may benefit by including anatomic region as a prognostic factor influencing management for each given stage. This has the potential to facilitate escalation or de-escalation of care when necessary. Additionally, future trials evaluating cutaneous melanoma would benefit from stratifying data into anatomic subsites as well as geographic distributions to further elucidate the cause of survival outcomes. 

## Figures and Tables

**Table 1:** Patient Demographics.

Characteristics	Total	Head and Neck	Trunk	Upper Extremity/Shoulder	Lower Extremity/Hip	*p*-Value	Effect Size
Cases (%)	178,892 (100)	38,335 (21.4)	60,312 (33.7)	47,292 (26.4)	32,953 (18.4)		
Mean Age (SD)	60.6 (16.7)	66.6 (16.6)	58.1 (16.0)	61.7 (15.9)	56.5 (16.8)	<0.001	
Age at Diagnosis						<0.001	0.1958
Age < 60 years	82,033 (45.9)	11,369 (29.7)	31,428 (52.1)	20,384 (43.1)	18,852 (57.2)		
Age ≥ 60 years	96,859 (54.1)	26,966 (70.3)	28,884 (47.9)	26,908 (56.9)	14,101 (42.8)		
Sex						<0.001	0.3026
Female	77,131 (43.1)	10,666 (27.8)	20,474 (33.9)	22,704 (48.0)	23,287 (70.7)		
Male	101,761 (56.9)	27,669 (72.2)	39,838 (66.1)	24,588 (52.0)	9666 (29.3)		
Race						<0.001	0.0465
Unknown	8554 (4.8)	1367 (3.6)	3348 (5.6)	2277 (4.8)	1562 (4.7)		
AI/AN	339 (0.2)	81 (0.2)	93 (0.2)	95 (0.2)	70 (0.2)		
Asian	1097 (0.6)	153 (0.4)	262 (0.4)	222 (0.5)	460 (1.4)		
Black	812 (0.5)	100 (0.3)	142 (0.2)	132 (0.3)	438 (1.3)		
White	168,090 (94.00)	36,634 (95.6)	56,467 (93.6)	44,566 (94.2)	30,423 (92.3)		
Region						<0.001	0.0211
Northeast	28,558 (16)	5355 (14)	10,119 (16.8)	7394 (15.6)	5690 (17.3)		
South	14,045 (7.9)	2995 (7.8)	4619 (7.7)	3787 (8)	2644 (8.0)		
Midwest	37,485 (21)	8182 (21.3)	12,890 (21.4)	10,007 (21.2)	6406 (19.4)		
West	98,804 (55.2)	21,803 (56.9)	32,684 (54.2)	26,104 (55.2)	18,213 (55.3)		
Insurance (2007+)						<0.001	0.024
Unknown	75,921 (42.4)	15,507 (40.5)	26,865 (44.5)	20,061 (42.4)	13,488 (40.9)		
None	2244 (1.3)	368 (1.0)	867 (1.4)	552 (1.2)	457 (1.4)		
Medicaid	3889 (2.2)	814 (2.1)	1203 (2.0)	987 (2.1)	885 (2.7)		
Insured (Includes Medicare)	96,838 (54.1)	21,646 (56.5)	31,377 (52)	25,692 (54.3)	18,123 (55)		

Column percentages are noted for patient demographics. AI/AN: American Indian & Alaskan Native. Asian: Asian and Pacific Islander. *p* < 0.05 is considered significant.

**Table 2:** Tumor Characteristics.

Characteristics	Total	Head and Neck	Trunk	Upper Extremity/Shoulder	Lower Extremity/Hip	*p*-Value	Effect Size
Characteristics							
AJCC Overall Stage						<0.001	0.0586
I	127,545 (71.3)	25,285 (66)	44,600 (73.9)	34,461 (72.9)	23,199 (70.4)		
II	20,889 (11.7)	5973 (15.6)	5559 (9.2)	5808 (12.3)	3549 (10.8)		
III	11,095 (6.2)	2024 (5.3)	3899 (6.5)	2365 (5.0)	2807 (8.5)		
IV	2651 (1.5)	741 (1.9)	944 (1.6)	457 (1.0)	509 (1.5)		
Unknown	16,712 (9.3)	4312 (11.2)	5310 (8.8)	4201 (8.9)	2889 (8.8)		
Ulceration						<0.001	0.0385
None	146,412 (81.8)	30,558 (79.7)	50,336 (83.5)	39,057 (82.6)	26,461 (80.3)		
Yes	21,869 (12.2)	5163 (13.5)	6627 (11)	5534 (11.7)	4545 (13.8)		
Unknown	10,611 (5.9)	2614 (6.8)	3349 (5.6)	2701 (5.7)	1947 (5.9)		
Mitoses						<0.001	0.0745
None	37,216 (20.8)	7332 (19.1)	13,664 (22.7)	9754 (20.6)	6466 (19.6)		
Yes	30,988 (17.3)	7241 (18.9)	9276 (15.4)	8531 (18)	5940 (18)		
Unknown	110,688 (61.9)	23,762 (62)	37,372 (62)	29,007 (61.3)	20,547 (62.4)		
Breslow’s Depth						<0.001	0.0459
0.0–1.00 Mm	116,853 (65.3)	22,773 (59.4)	41,781 (69.3)	31,235 (66)	21,064 (63.9)		
1.01–2.00 Mm	24,632 (13.8)	5462 (14.2)	7601 (12.6)	6740 (14.3)	4829 (14.7)		
2.01–4.00 Mm	14,493 (8.1)	3765 (9.8)	4033 (6.7)	3836 (8.1)	2859 (8.7)		
>4.00 Mm	10,705 (6.0)	3006 (7.8)	3122 (5.2)	2609 (5.5)	1968 (6.0)		
Unknown	12,209 (6.8)	3329 (8.7)	3775 (6.3)	2872 (6.1)	2233 (6.8)		
Histology						<0.001	0.1543
Malignant Melanoma NOS	89,882 (50.2)	17,907 (46.7)	31,161 (51.7)	23,948 (50.6)	16,866 (51.2)		
Nodular Melanoma	12,422 (6.9)	2984 (7.8)	3811 (6.3)	3565 (7.5)	2062 (6.3)		
Lentigo Maligna Melanoma	12,093 (6.8)	6997 (18.3)	2028 (3.4)	2555 (5.4)	513 (1.6)		
Superficial Spreading	54,968 (30.7)	7844 (20.5)	20,991 (34.8)	14,930 (31.6)	11,203 (34.0)		

Column percentages are noted for patient demographics. *p* < 0.05 is considered significant.

**Table 3:** Survival Outcomes Related to Clinicopathologic Characteristics.

Characteristics	5-Year Overall Survival			
	Head and Neck	Trunk	Upper Extremities/Shoulder	Lower Extremities/Hip
Ulceration				
None	76%	88%	87%	92%
Yes	46%	54%	56%	54%
Mean Difference, 95% CI	−30.0, 26.9–33.1	−34.0, 31.6–36.4	−31.0, 28.4–33.6	−38.0, 35.1–40.9
Mitoses				
None	90%	90%	90%	93%
Yes	63%	76%	77%	76%
Mean Difference, 95% CI	NA	NA	NA	NA
Slnb				
Yes	74%	82%	82%	85%
No	72%	86%	85%	88%
Mean Difference, 95% CI	+2.0, 0.1–3.9	−4.0, 2.9–5.1	−3.0, 1.8–4.2	−3.0, 1.7–4.3
Surgery				
Yes	74%	85%	84%	87%
No	59%	77%	76%	79%
Mean Difference, 95% CI	+15.0, 10.0–20.2	+8.0, 4.7–11.7	+8.0, 4.1–12.3	+8.0, 3.7–13.0

Abbreviations: SLNB, Sentinel Lymph Node Biopsy.

**Table 4:** Univariate and Multivariate Cox Hazard Ratio Analysis Identifying Characteristics Associated with Death.

Variable	Univariate Analysis: Hazard Ratio (95% CI)	Multivariate Analysis: Adjusted Hazard Ratio (95% CI)
Age Category		
Age < 60 years	Reference	Reference
Age ≥ 60 years	4.78 (4.64–4.92)	3.79 (3.68–3.89)
Sex		
Female	Reference	Reference
Male	1.74 (1.70–1.78)	1.32 (1.27–1.38)
Anatomic Region		
Trunk	Reference	Reference
Head and Neck	1.90 (1.85–1.96)	1.30 (1.27–1.37)
Upper Extremity/Shoulder	1.06 (1.03–1.10)	0.97 (0.92–1.03)
Lower Extremity/Hip	0.86 (0.82–0.89)	0.88 (0.82–0.94)
AJCC Overall Stage		
I	Reference	Reference
II	3.88 (3.77–4.00)	1.27 (1.15–1.39)
III	4.82 (4.65–4.99)	2.67 (2.42–2.95)
IV	22.45 (21.42–23.53)	6.00 (5.34–6.76)
Surgical Management		
None	Reference	Reference
Local Tumor Excision	0.54 (0.51–0.57)	0.60 (0.53–0.68)
Gross Excision < 1 mm	0.32 (0.30–0.33)	0.38 (0.34–0.42)
Gross Excision > 1 mm	0.41 (0.40–0.43)	0.40 (0.36–0.45)
Surgery NOS	0.95 (0.82–1.10)	0.51 (0.29–0.91)
SLNB		
None	Reference	Reference
Yes	1.03 (1.00–1.06)	0.55 (0.52–0.58)
Mitoses		
None	Reference	Reference
Yes	3.09 (2.91–3.27)	1.29 (1.20–1.40)
Ulceration		
None	Reference	Reference
Yes	4.14 (4.03–4.24)	1.81 (1.71–1.91)
Breslow’s Depth		
0.0–1.00 mm	Reference	Reference
1.01 0 2.00 mm	1.95 (1.88–2.02)	1.81 (1.63–1.94)
2.01–4.00 mm	3.93 (3.81–4.07)	2.33 (2.11–2.57)
>4.0 mm	7.36 (7.13–7.61)	3.19 (2.89–3.52)
Histological Type		
Superficial Spreading Melanoma	Reference	Reference
Lentigo Maligna Melanoma	2.25 (2.14–2.36)	1.26 (1.15–1.37)
Nodular Melanoma	4.15 (4.01–4.30)	1.20 (1.13–1.29)
Malignant Melanoma NOS	1.52 (1.39–1.66)	1.06 (1.01–1.12)
Desmoplastic Melanoma, Malignant	1.14 (0.29–4.57)	0.96 (0.84–1.11)
Spindle Cell Melanoma	1.76 (1.61–1.91)	0.94 (0.83–1.08)

Abbreviations: SLNB, Sentinel Lymph Node Biopsy.

**Table 5:** Management and Survival.

Characteristics	Total	Head and Neck	Trunk	Upper Extremity/Shoulder	Lower Extremity/Hip	*p*-Value	Effect Size
Sentinel Lymph Node Biopsy						<0.001	0.0802
None	119,253 (66.7)	27,171 (70.9)	40,989 (68.0)	30,746 (65.0)	20,347 (61.7)		
Yes	45,062 (25.2)	7647 (19.9)	14,598 (24.2)	13,078 (27.7)	9739 (29.6)		
Unknown	14,577 (8.1)	3517 (9.2)	4725 (7.8)	3468 (7.3)	2867 (8.7)		
Surgical Management						<0.001	0.0369
None	7160 (4.0)	1848 (4.8)	2324 (3.9)	1641 (3.5)	1347 (4.1)		
Local Tumor Excision	23,747 (13.3)	5078 (13.2)	8403 (13.9)	6140 (13.0)	4126 (12.5)		
Gross Excision < 1 cm Margins	85,457 (47.8)	19,678 (51.3)	28,100 (46.6)	22,910 (48.4)	14,769 (44.8)		
Gross Excision > 1 cm Margins	61,386 (34.3)	11,368 (29.7)	21,131 (35.0)	16,359 (34.6)	12,528 (38.0)		
Surgery NOS	496 (0.3)	178 (0.5)	141 (0.2)	97 (0.2)	80 (0.2)		
Unknown	646 (0.4)	185 (0.5)	213 (0.4)	145 (0.3)	103 (0.3)		
Mortality							
2-year OS	91%	86%	92%	92%	93%		
5-year OS	80%	71%	83%	82%	85%		

Column percentages are noted for patient demographics. *p* < 0.05 is considered significant.

## Data Availability

All data included is available in the Surveillance, Epidemiology, and End Results Program (SEER) Database.
